# Supplementary material for: An integrated single cell and spatial transcriptomic map of human white adipose tissue
Source: Nat Commun. 2023 Mar 15;14:1438. doi: 10.1038/s41467-023-36983-2 (PMC10017705; doi:10.1038/s41467-023-36983-2)
Supplement: Supplementary file 1 — Supplementary Information [file 41467_2023_36983_MOESM1_ESM.pdf]

Supplementary Information

An integrated single cell and spatial transcriptomic map of human white adipose tissue

Lucas Massier[1], Jutta Jalkanen[1], Merve Elmastas[1], Jiawei Zhong[1], Tongtong Wang[2], Pamela A. Nono Nankam[3], Scott Frendo-Cumbo[1], Jesper Bäckdahl[1], Narmadha Subramanian[1], Takuya Sekine[4], Alastair G. Kerr[1], Ben T. P. Tseng[1], Jurga Laurencikiene[1], Marcus Buggert[4], Magda Lourda[4,5], Karolina Kublickiene[6], Nayanika Bhalla[7], Alma Andersson[7], Armand Valsesia[8], Arne Astrup[9], Ellen E. Blaak[10], Patrik L. Ståhl[7], Nathalie Viguerie[11,12], Dominique Langin[11,12,13,14], Christian Wolfrum[2], Matthias Blüher[3,15], Mikael Rydén[1*], Niklas Mejhert[1*]

[1] Department of Medicine Huddinge (H7), Karolinska Institutet, Karolinska University Hospital Huddinge, SE-141 83, Huddinge, Sweden.

[2] Laboratory of Translational Nutrition Biology, Institute of Food, Nutrition and Health, Department of Health Sciences and Technology, ETH Zurich, Schwerzenbach, Switzerland.

[3] Helmholtz Institute for Metabolic, Obesity and Vascular Research (HI-MAG) of the Helmholtz Zentrum München at the University of Leipzig and University Hospital Leipzig, Germany.

[4] Center for Infectious Medicine, Department of Medicine Huddinge (H7), Karolinska Institutet, Karolinska University Hospital Huddinge, SE-141 52, Huddinge, Sweden.

[5] Childhood Cancer Research Unit, Department of Women's and Children's Health, Karolinska Institutet, SE-171 77, Stockholm, Sweden

[6] Department of Clinical Science, Intervention & Technology (CLINTEC), Unit of Renal Medicine, Karolinska Institutet, Karolinska University Hospital Huddinge, SE-141 86, Huddinge, Sweden.

[7] Science for Life Laboratory, Department of Gene Technology, KTH Royal Institute of Technology, SE-171 65, Solna, Sweden.

[8] Department of Metabolic Health, Nestle Institute of Health Sciences, Nestle Research, Lausanne, Switzerland

[9] Department of Obesity and Nutritional Sciences, The Novo Nordisk Foundation, Hellerup, Denmark.

[10] Department of Human Biology, NUTRIM School of Nutrition and Translational Research in Metabolism, Maastricht University Medical Centre(+), Maastricht, the Netherlands.

[11] Institute of Metabolic and Cardiovascular Diseases (I2MC), Institut National de la Santé et de la Recherche Médicale (Inserm), Université Toulouse III - Paul Sabatier (UPS), Université de Toulouse, UMR1297, Toulouse, France.

[12] Franco-Czech Laboratory for Clinical Research on Obesity, Third Faculty of Medicine, Charles University, Prague and Université Toulouse III - Paul Sabatier (UPS), Toulouse, France

[13] Laboratoire de biochimie, Centre Hospitalier Universitaire de Toulouse, Toulouse, France

[14] Institut Universitaire de France (IUF), Paris, France

[15] Medical Department III - Endocrinology, Nephrology, Rheumatology, University of Leipzig Medical Center, Germany.

Correspondence and requests should be addressed to Niklas Mejhert (niklas.mejhert@ki.se) and Mikael Rydén (mikael.ryden@ki.se).

* These authors contributed equally.

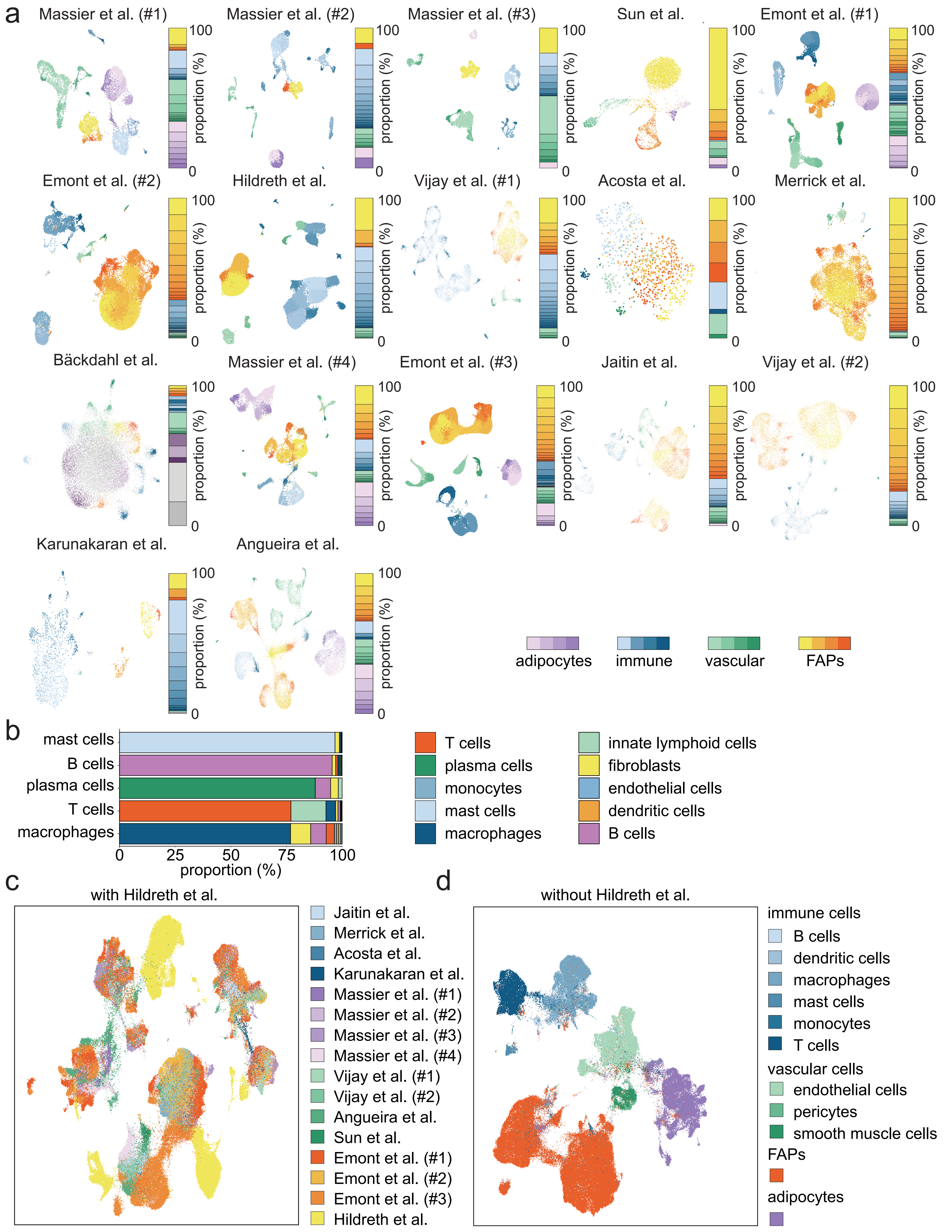

Supplementary Figure 1. Detailed comparison of individual studies (related to main Figure 1).
a. Multiple UMAPs and stacked bar charts from the studies analyzed herein.
b. Immune models from CellTypist applied to the immune cell class identified herein.
c-d. Single-cell annotation using variational inference (scANVI) analysis c) including or d) excluding Hildreth et al.

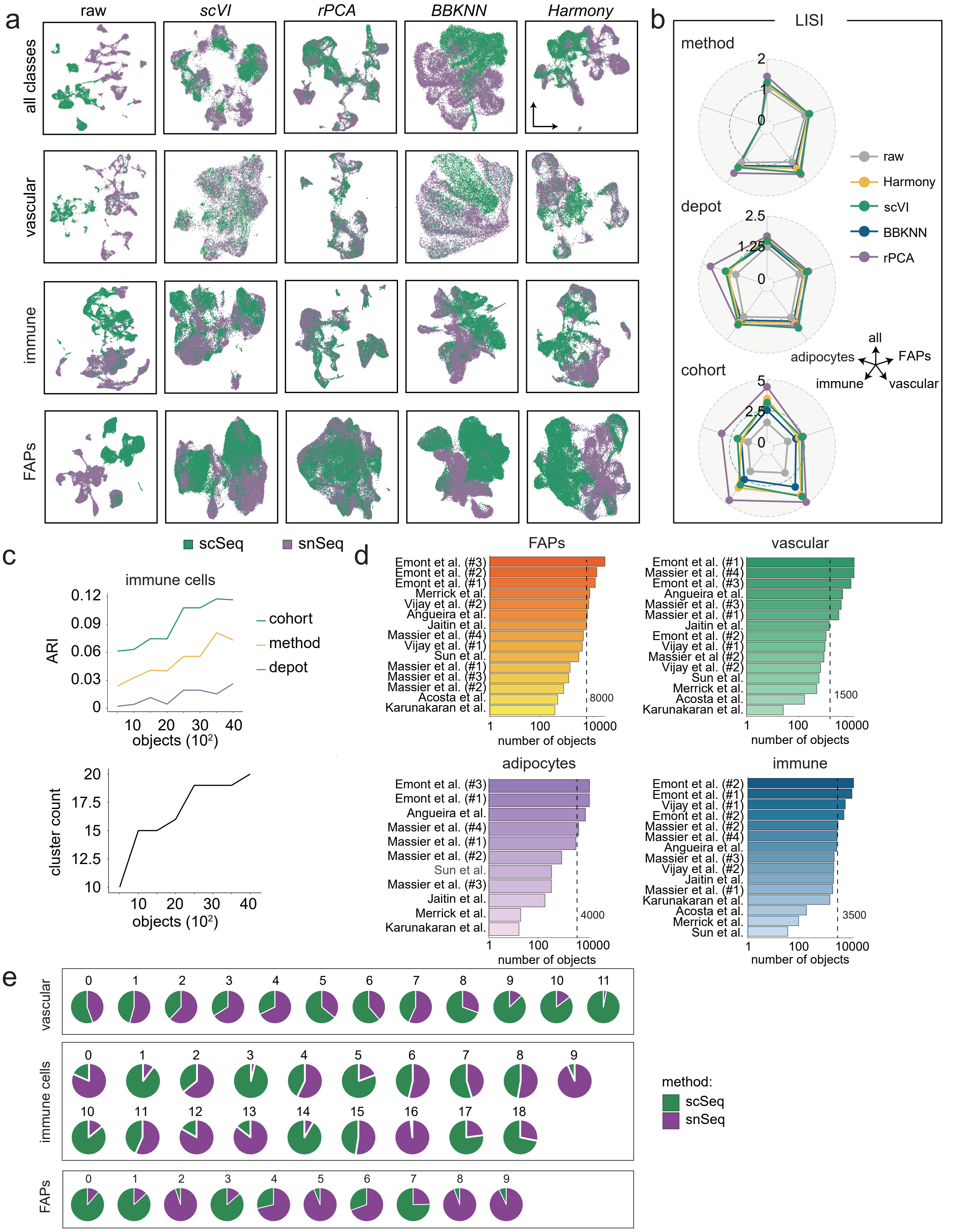

Supplementary Figure 2. Optimization of integration (related to main Figure 1).
a. Multiple UMAP plots of unintegrated (raw) and integrated data using the indicated approaches (details in Methods). Upper panels show results using data from all cell classes, while the lower panels display results for individual cell classes. Colors indicate method: single-cell (scSeq, green) and single-nucleus (snSeq, purple) sequencing. b. Local inverse Simpson's index (LISI) for raw or integrated data using the indicated methods displayed according to method, depot and cohort. c. A representative example displaying how the adjusted Rand index (ARI, upper panel) and number of immune cell clusters (lower panel) vary depending on the number of included objects. d. Number of objects included from each study per cell class. Maximum cut-off is highlighted by the vertical dashed line. e. Proportion (%) of scSeq or snSeq objects in the indicated cell classes.

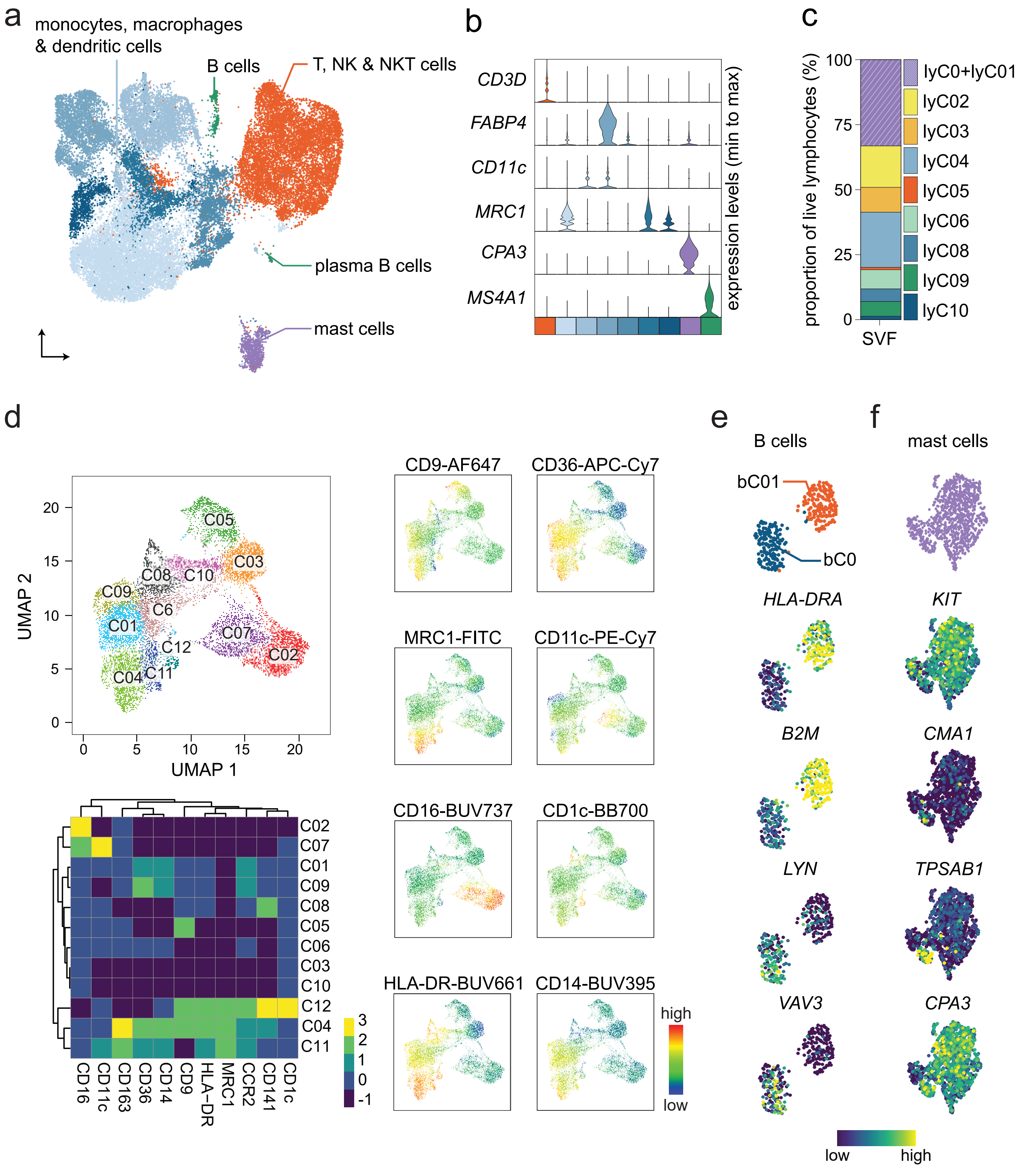

Supplementary Figure 3. Flow-cytometry validation of immune cells (related to main Figure 2).

a. Immune cells broadly separate into macrophages, monocytes, T, NKT, NK, B, plasma B, and mast cells.

b. Selected marker genes for each immune cell group. Colors are matched with those in panel a.

c. Proportions of indicated lymphoid clusters using flow-cytometry of white adipose tissue (WAT)-resident immune cells from 10 subjects. LyC0 and lyC01 were defined as CD3+, CD4+, non-naïve (CCR7+, CD45RA+) T cells, lyC02 and lyC03 were defined as CD3+, CD8+, non-naïve (CCR7+, CD45RA+), CD57- or CD57+ T cells, respectively. LyC04 was gated on CD3+, CD4+, CCR7+, CD45-RA+ T cells; lyC05 CD3+, CD56+, CD16+ NKT cells; lyC06 CD3-, CD56+, CD16+ NK cells; lyC08 CD3+, CD4+, CD127-, CD25+, FoxP3+ Tregs; lyC09 CD3-, CD56+, CD16- NK cells; and lyC10 CD3+, CD4+, non-naïve (CCR7+,CD45RA+), CD69+, HLA-DR+ T cells.

d. UMAP of WAT-resident myeloid cells gated as live, single, CD45+, dump-(CD3, CD19, CD56, CD304) cells (upper left). Results were generated by flow-cytometry from 10 samples. Main surface markers per myeloid cell cluster are highlighted in the adjacent UMAPs (right) and the heatmap (lower left).

e. B cells separate into activated (bC01) and non-activated (bC0) states based on multiple marker genes including the indicated ones.

f. Same as panel e, but for mast cells. No clear subclusters were obtained.

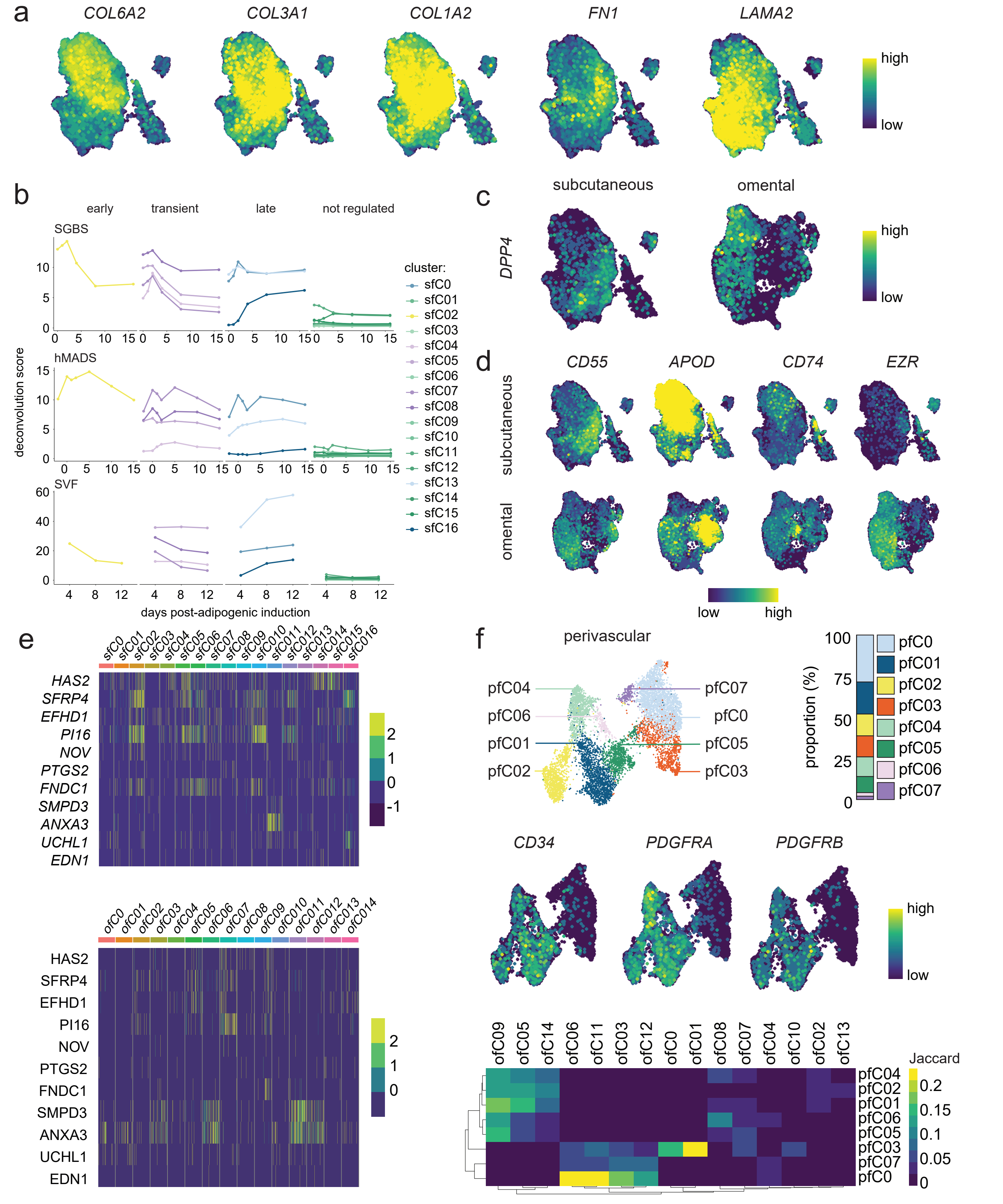

Supplementary Figure 4. Depot-specific differences in FAP signatures (related to main Figure 4).
a. Multiple UMAPs showing the expression of selected genes encoding extracellular matrix proteins in subcutaneous FAPs.
b. Expression of clusters sfC0-16 in Simpson-Golabi-Behmel Syndrome (SGBS), (human multipotent adipose-derived stem (hMADS), and primary cells isolated from human stromal vascular fraction (SVF) during in vitro differentiation.
c-d. Multiple UMAPs displaying the expression of marker genes in subcutaneous (sc) and omental (om).
e. Expression of fibro-inflammatory progenitor marker genes in subcutaneous (upper panel) and omental (lower panel) FAPs.
f. A UMAP and a stacked bar chart displaying the proportion (%) of cell clusters in perivascular WAT (upper panels). Expression of selected genes shown in UMAPs (mid panels). Comparisons of similarities between omental and perivascular FAP marker genes (lower panels).

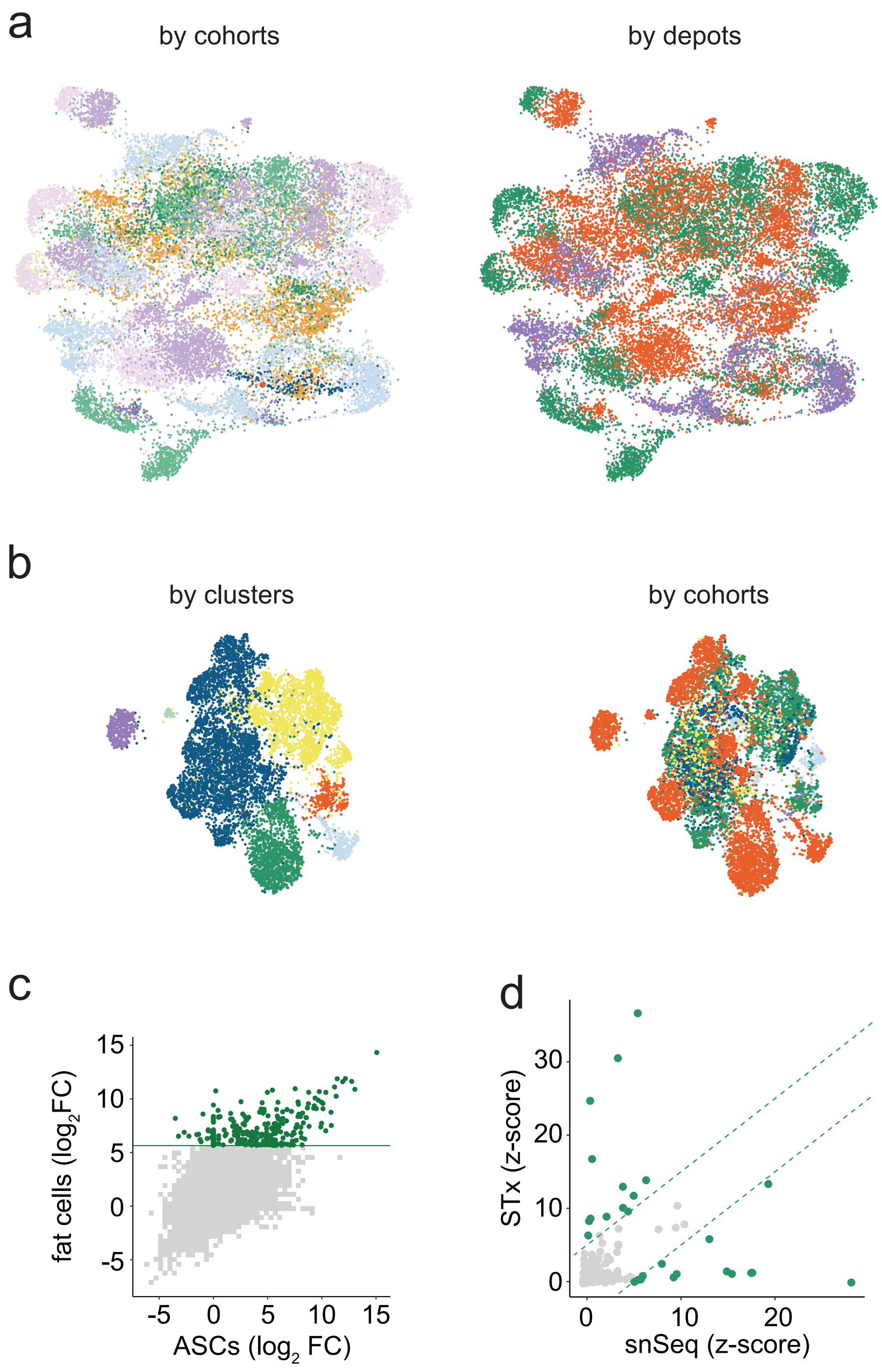

Supplementary Figure 5. Adipocytes separate by cohort and depot after integration (related to main Figure 5).

a. Two UMAPs of adipocyte data from all available studies highlighting cohorts (left panel) and depots (right panel).
b. Two UMAPs of adipocyte data from all available studies highlighting clusters (left panel) and cohorts (right panel) for subcutaneous data only.
c-d. Selection of c) adipocyte-marker genes (vs. adipose-derived stem [ASC] cells) and d) marker genes discordant for spatial transcriptomics (STx) and single-nucleus sequencing (snSeq) data. For details on selection, see Methods.

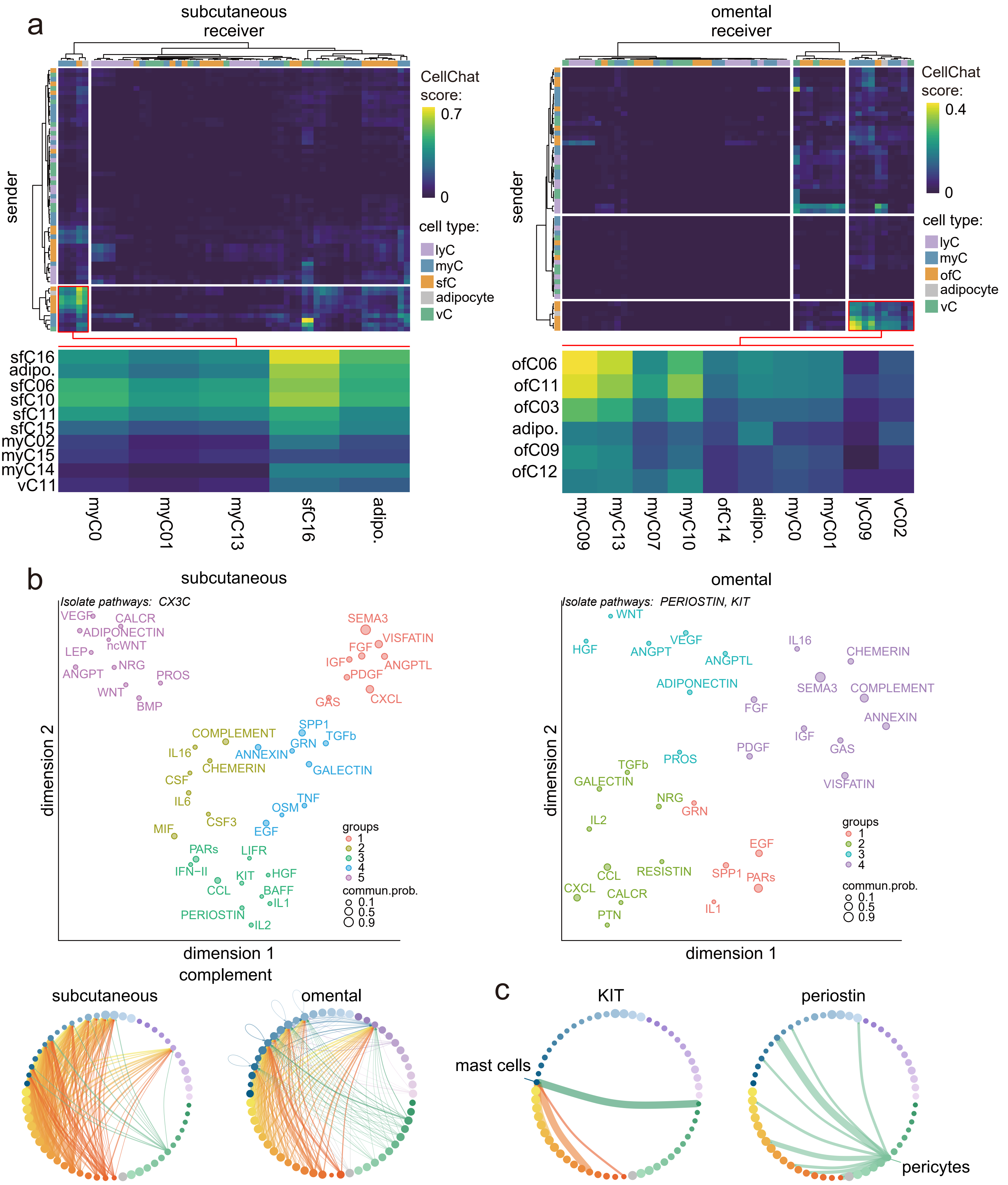

Supplementary Figure 6. Differences in ligand-receptor interactions between subcutaneous and omental WAT (related to main Figure 6).

a. Heatmaps indicating subcutaneous (left panel) and omental (right panel) white adipose tissue (WAT) CellChat scores where senders (rows) and receivers (columns) are displayed. The indicated red boxes are magnified in the lower panels.

b. Analyzed pathways were projected into a two-dimensional manifold by functional similarity using CellChat. Groups represent pathways of similar signaling directions, i.e., group 2 in subcutaneous and group 4 in omental WAT send signals from FAPs to myeloid. As a representative example signaling within the complement pathway is shown (lower panel).

c. Predicted cell-cell communication with the KIT and Periostin pathways for subcutaneous WAT. Lines indicate interactions between cell types where the strength is proportional to the line width and the color defines the sending subpopulation.

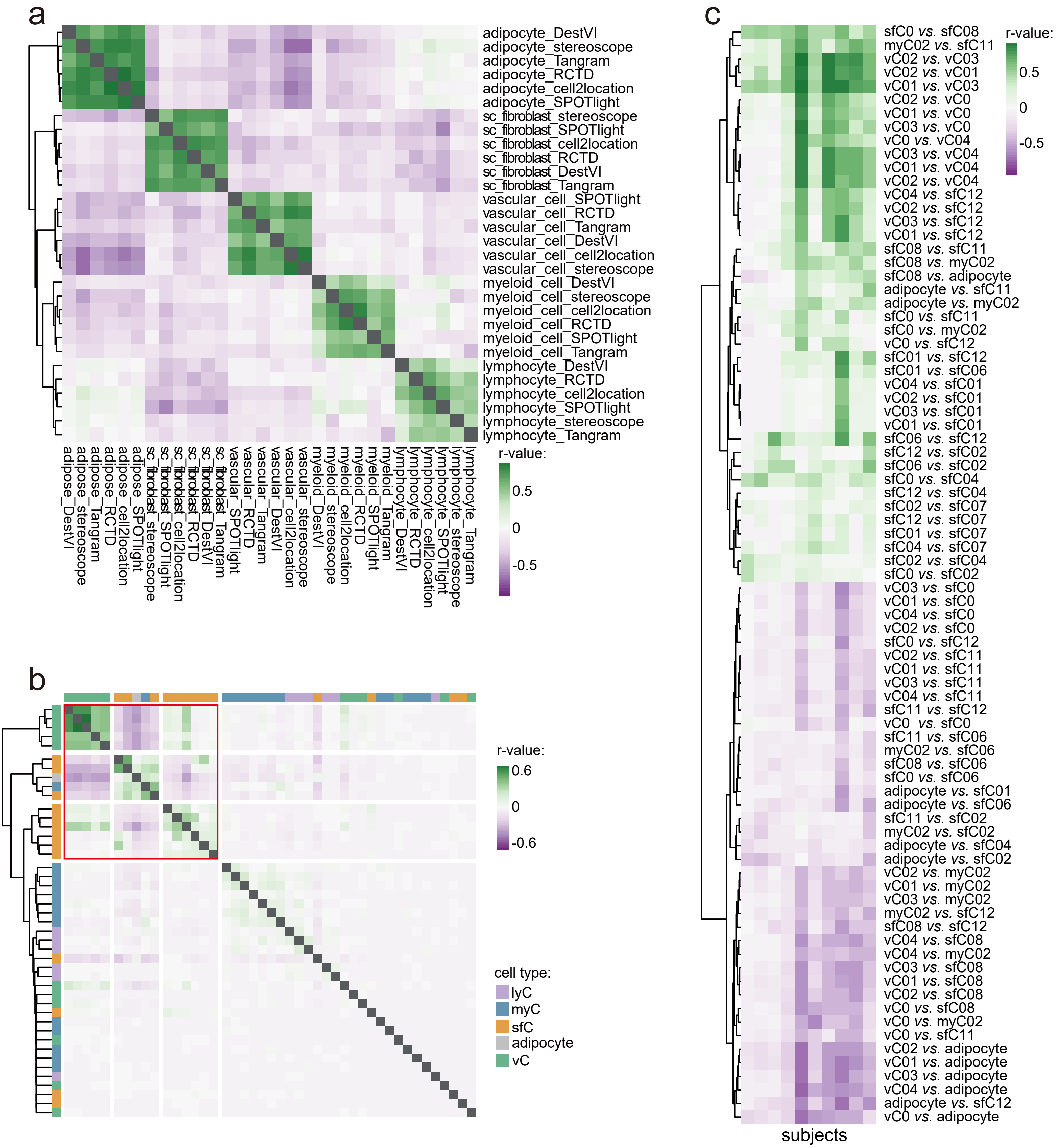

Supplementary Figure 7. Spot-based deconvolution of spatial transcriptomics data (related to main Figure 7). a-c. Heatmaps for a) the indicated deconvolution tools for the different cell classes, and for the pair-wise within-spot associations between b) cellular subpopulations and c) across all ten subjects for selected cells. In panel b, the area marked in red is magnified in main Figure 7d. In panel c, only correlations with an r-value >0.1 are included.

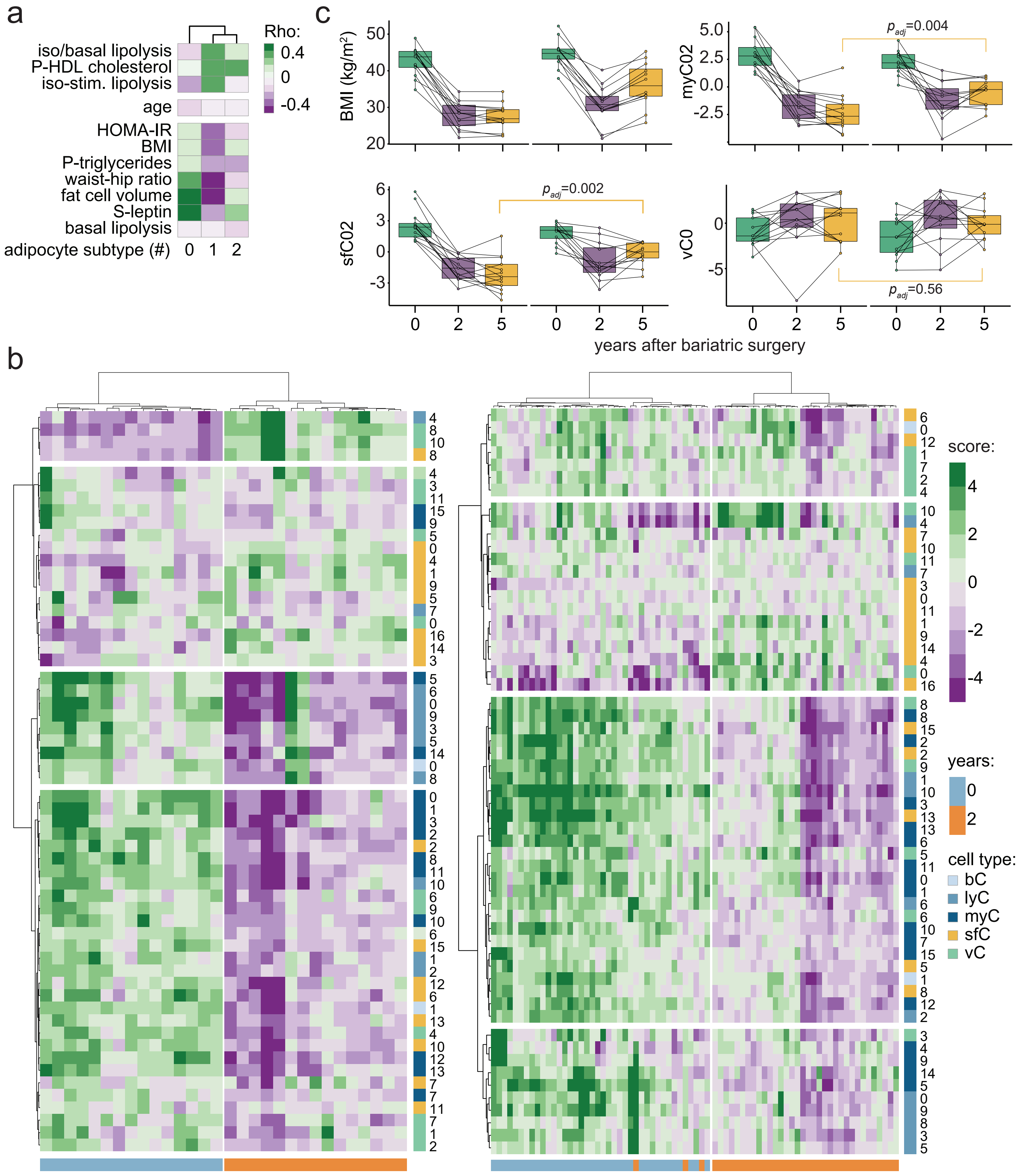

Supplementary Figure 8. Deconvolution of bulk transcriptomic data in weight loss and regain (related to main Figure 8).
a. Heatmap for the indicated clinical/WAT measures following deconvolution of the bulk transcriptomic data using spatial (STx) marker genes for adipocyte subtype #0 (AdipoLEP), #1 (AdipoPLIN) and #2 (AdipoSAA), respectively. Analyses were performed as in main Figure 8b.
b. Heatmaps detailing cell type deconvolution scores of subcutaneous white adipose tissue before and two years after bariatric surgery in Petrus et al.55 (left panel) and Kerr et al.54 (right panel).
c. Body mass index (upper left panel) and representative examples of cell type deconvolution scores before, two and five years after bariatric surgery in the study from Kerr et al.54 For each panel, data is subdivided according to subjects displaying weight stability (left part) and weight regain (right part) between the two and five year follow-ups. p values were calculated by two-sided Student's t test comparing differences at five years between weight stability (n=13) and weight regain (n=12) and boxplots are presented as interquartile range plus median and Tukey whiskers with individual, paired data points.

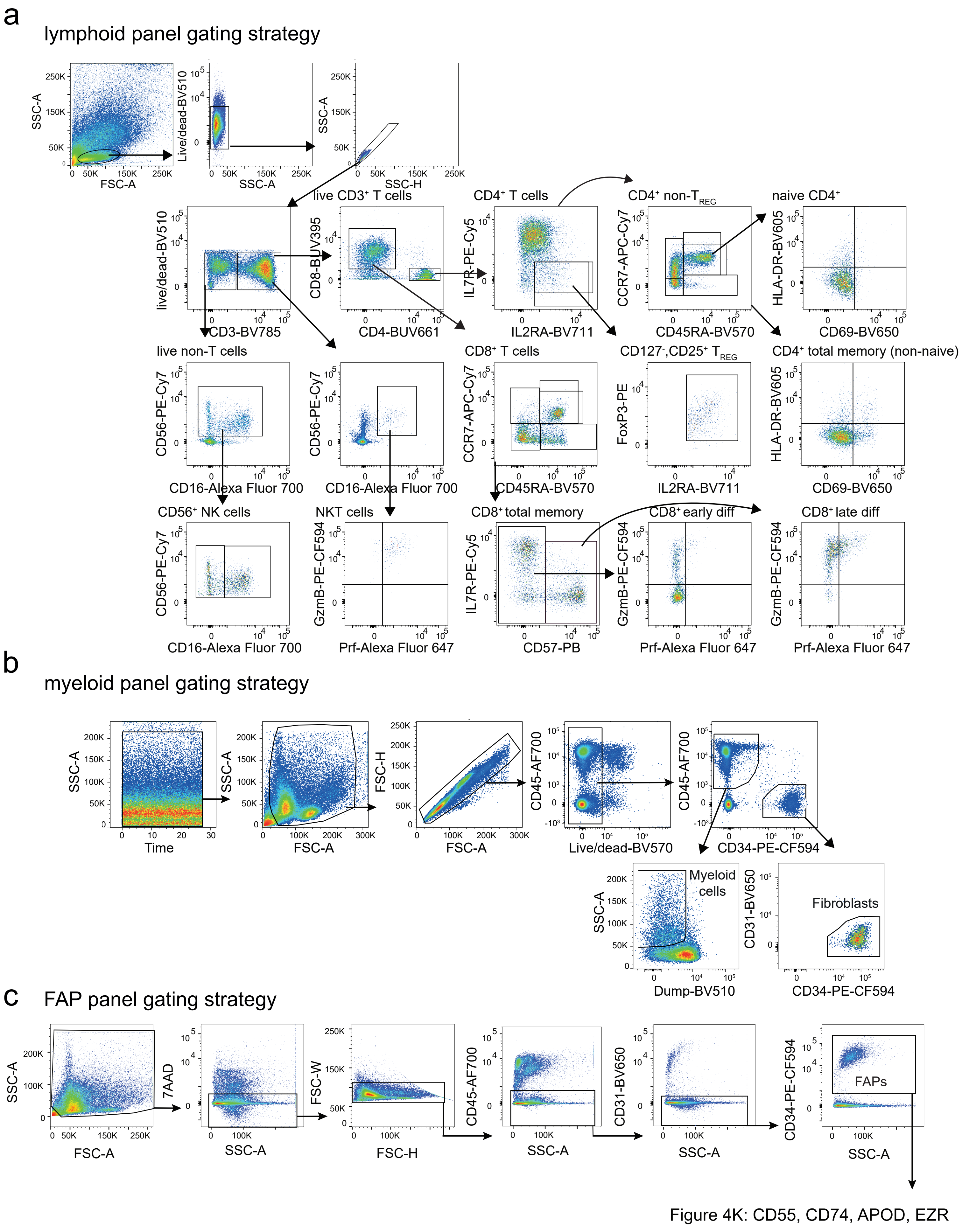

Supplementary Figure 9. Gating strategies (related to main Figures 2 and 4).
a-c. Flow-cytometry gating strategies for a) lymphoid, b) myeloid cells and c) fibroblast and adipogenic progenitors (FAPs).

**Supplementary Table 1. Details on datasets included in the meta-analysis.**					
**Publication**	**Method**	**Illumina platform**	**Collection**	**Data source**	**Reference**
Sun et al.	snSeq	NovaSeq 6000	Switzerland	E-MTAB-9199	Nature 587, 98-102 (2020)
Hildreth et al.	scSeq	NovaSeq 6000S2	USA	GSE155960, GSE156110	Nat Immunol 22, 639-653 (2021)
Vijay et al.	scSeq	NovaSeq 6000	Canada	GSE136230	Nat Metab 2, 97-109 (2020)
Acosta et al.	scSeq	HiSeq 2000	Sweden	Communication with author	Stem Cell Res Ther 8, 250 (2017)
Merrick et al.	scSeq	HiSeq 2500	USA	GSE128889	Science 364(2019)
Karunakaran et al.	scSeq	NovaSeq S2	Canada	GSE151889	Nat Metab 2, 1113-1125 (2020)
Emont et al.	snSeq, scSeq	NextSeq 500	USA	E-MTAB-6677, GSE135134, GSE128889	Nature 603, 926-933 (2022)
Jaitin et al.	scSeq	NextSeq 500	USA	GSE128518	Cell 178, 686-698 e614 (2019)
Angueira et al.	snSeq	NovaSeq 6000	USA	GSE164528	Nat Metab 3, 469-484 (2021)
Bäckdahl et al.	STx	NovaSeq 6000	Sweden	https://doi.org/10.17632/3bs5f8mvbs.1	Cell Metabolism 33, 1–14 (2021)

Supplementary Table 2: Confidence score and references for cluster assignment.				
**Cluster**	**Cell Annotation**	**Marker**	**PMID**	**Confidence**
**Lymphoid cells**				
lyC0	CD4 Th1	*CD4*	34193602	high
		STAT4	30714682	
		HLA-DR	22474485	
lyC01	CD4 TRM	*CD4*	34671115	high
		CD69		
		CD44	29262342	
		SELL (CD62L)- and *CCR7-*	29221731	
lyC02	Early-diff CD8	*CD8*	35677061	high
		IFNG	35477960	
lyC03	Late-diff CD8	*CD8*	12766758	
		GZMB	12001998	
		PRF1		
		GNLY		
lyC04	Naive/early-diff CD4	*CD4*	30559746	medium
		CCR7	22990666	
		SELL	22474485	
		STAT4	35092032	
		LEF1		
lyC05	NKT	*NCAM1*	11359797	high
		CD56	15238416	
		GZMB		
lyC06	CD16+ NK cells	*FCG3RA /CD56*	11698225	high
		KLRF1	10671213	
		NKG7	32839609	
		CD3- (NK cells lack *CD3*)	11698225	
			15368309	
lyC07	Unknown	*N/A*		
lyC08	Naive /Treg CD4	*CD25 (IL2RA)*	26122357	medium
		CD127 (IL7R)	21635972	
		CD4		
		LEF1	35092032	
lyC09	CD56+CD16- NK-cells	*CD56*	11698225	high
		CYRIB		
lyC10	CD4 T cells	*CD4*	28280496	high (phenotype low)
		HLA-DR	28167943	
		LAMP1 (CD107)		
		CD69		
**Myeloid cells**				
myC0	M2-like macrophage	*MRC1*	35480482	high
		F13A1	33221826	
		SLC9A9		
		RBPJ	26874522	

myC01	M2-like macrophage	*MRC1*	35480482	high
		RBPJ	26874522	
		SLC9A9		
		F13A1	33221826	
myC02	Lipid associated macrophage (LAM)	*TREM2*	31257031	high
		CD9	29760084	
		FABP4		
		CD68	34794433	
myC03	Dendritic cell type 2 (cDC2)	*CD1c*	33101275	high
		CD74		
		HLA-DR	31474513	
		CLEC10A	31668803	
		TIMP1		
myC04	M2-like macrophage	*LYVE1*	18227385, 17272806	high
		FOS	31178859	
		LGMN	25205715	
Comments: LYVE1high macrophages indicated role in tissue support and angiogenesis.				
myC05	Non-classical monocyte	*FCGR3A=CD16*	21653326	high
		LST1		
		TCF7L2	34331874	
		LYST		
myC06	M1-/M2-like macrophage	*ITGAX (CD11c)*	25035950	high
		CTSB	17200717	
		ITGAM (CD11b)	20357360	
myC07	M2-like macrophage	*FN1*	35202577	high
		MARCO	25035950	
		TIMD4	34381461	
		FCGR3A	18227385	
Comment: Expressing genes related to vasculature-associated macrophages with high scavenging capacity.				
myC08	M2-like macrophage	*TIMP1*	7593617	low
		C3	22194611	
		IFITM3	33364194	
		S100A10	31615030	
		MT1E	17322100	
Comment: Gene expression profile indicative of cell-matrix interaction and ECM remodeling				
myC09	M2-like macrophage	*MRC1*	35480482	high
		TGFBI	25242226	
		ABCA1		
		PPARG	18522830	

myC10	Metabolic-regulated macrophage (Mme)	*MSR1*	32066456	high
		CD36	25242226	
		PPARG		
		ITGAX (CD11c)		
		PLIN2		
myC11	M2-like macrophage	*C1QA*	34331874, 35288093	medium
		C1QB		
		C1QC		
		SEPP1	33033253	
		RNASE1		
Comment: Resembles C1Qhi macrophage with M2-like gene expression.				
myC12	M2-like macrophage	*C3*	22194611	low
		STAT3	30015355	
		FKBP5	29241532	
		PTPRG	16896153	
myC13	M2-like macrophage	*MRC1*	35480482	high
		MERTK	23023392	
		SQSTM1	18083104	
		MARCH-proteins	32973799	
Comment: M2 macrophages with high levels of E3 ligases indicating increased autophagy and inhibition of inflammasome signaling.				
myC14	Classical monocyte	*S100A8*	21653326	high
		S100A9		
		CD14		
		LYZ		
		VCAN	34331874	
		CCL3	31543877	
myC15	Redox-regulatory metabolic macrophages (MOX)	*HMOX*	25600948	high
		FTH1	25600948	
		IL1B	29891687	
		TNF		
		TXNRD1	30229891	
		NFE2L2 (NRF2)	20651288	
		HSPA5	20514049	
		CCL2	19404946	
Comment: Both Ox-phospholipid induced proinflammatory and antioxidant phenotype; also expressing iron handling-related genes.				
**Vascular cells**				
vC0	BEC - Capillary	*CD36, BTNL9*	34030460	high
vC01	BEC - Vein	*EPHB4, ACKR1*	30154011, 28526034	high

vC02	BEC - Artery	*EFNB2*	11161569	high
vC03	BEC - Capillary/venous	*CA4*	34030460	high
vC04	Pericyte	*PDGFRB, CSPG4, RGS5*	21839917	high
vC05	BEC	*CFD, PDGFRA*	33846639	high, (subtype low)
vC06	LEC	*PROX1, LYVE1*	10499794	high
vC07	VSMC	ACTA2, MYH11, TAGLN	31340668	high
vC08	BEC	*TIMP1*	7517405	high, (subtype low)
vC09	BEC	*FTL*		high, (subtype low)
vC010	Unknown	*N/A*		low
vC011	BEC - Vein	*ACKR1*	28526034	medium
Comparing enriched marker genes in the respective cluster to a manually curated list of specific endothelial cell subtype markers from the publications PMID: 34030460 and 32059779				
Cluster	Capillary (n=35)	Artery (n=28)	Vein (n=32)	
vC0	14	2	5	
vC01	1	2	18	
vC02	4	19	1	
vC03	8	2	9	
vC05	5	5	2	
vC08	2	1	1	
vC09	2	0	0	
vC011	3	1	8	
List of included genes				
Capillary: *MFDSD2A, AQP7, KDR, RGCC, CA4, SPARC, SGK1, PRX, CYB5A, ACVRL1, ADGRF5, ADGRL2, F2RL3, INFGR1, VIPR1, ADRB1, ARHGAP6, IFI27, PREX1, PRKCE, SH2D3C, SORBS1, EMP2, ITGA1, SLC9A3R2, AFF3, MEIS1, CD36, GPIHBP1, FCN3, BTNL9, BTNL8, CD14, IL7R, IL18R1*				
Artery: *CYTL1, GKN3, EFNB2, SOX17, BMX, SEMA3G, HEY1, LTBP4, GJA5, GJA4, CLDN10, FBLIM1, FBLN2, FBLN5, MGP, FN1, LTBP1, SERPINE2, CPAMD8, CXCL12, EFBN2, VEGFA, NOS1, DKK2, DLL4, SOX5, HES4, PRDM16*				
Vein: *LCN2, SLC38A5, BGN, PTGS1, CH25H, BST2, CAR4, VCAM1, SELP, SELE, ACKR1, NR2F2, ADAMTS9, IGFBP7, HDAC9, RORA, ACTN1, LDLRAD3, LRRC1, EBF1, EBF3, MEOX1, MEOX2, ZNF385D, TACR1, ROBO1, CYSLTR1, CPXM2, MMP16, PDE7B, PDE2A, SPRY1*				

Abbreviations: BEC; Blood endothelial cell, LEC; Lymphatic endothelial cell, VSMC; vascular smooth muscle cell

**Supplementary Data Table 3. Datasets used for clinical correlations in Figure 8.**					
**Publication**	**Method**	**Platform**	**Collection**	**Data source**	**Reference**
Arner, E. et al.	Microarray	GeneChip Human Gene 1.0 ST Array	Sweden	GSE25402	Diabetes 61, 1986-1993 (2012)
Arner, P. et al.	Microarray	Clariom D array	Sweden	GSE113080	Cell Metabolism 28, 45-54 (2018)
Kerr, A. et al.	Microarray	Clariom D array	Sweden	GSE199063	J Intern Med 288, 219-233 (2020)
Arner, P. et al.	Microarray	GeneChip Human Transcriptome Array 2.0	Sweden	GSE76399	Diabetologia 59, 2393-2405 (2016)
Petrus, P. et al.	Microarray	Affymetrix Human Gene 1.1 ST array	Sweden	GSE59034	Cell Rep 25, 551-560 (2018).
Krieg, L et al.	Microarray	HumanHT - 12	Germany	Suppl. Table 7	Gut 71, 2179-2193 (2022).
Imbert, A. et al.	RNASeq	HiSeq 2500	Netherlands, Denmark, United Kingdom, Greece, Bulgaria, Germany, Spain, and Czech Republic	GSE141221	J Clin Endocrinol Metab 107, e130-e142 (2022).
Armenise, C. et al.	RNASeq	HiSeq 2000	Netherlands, Denmark, United Kingdom, Greece, Bulgaria, Germany, Spain, and Czech Republic	GSE95640	Am J Clin Nutr 106, 736-746 (2017).

Supplementary Table 4. Fluorochrome conjugated antibodies used in the flow cytometric analysis.

Myeloid panel

**Fluorochrome**	**Marker**	**Clone**	**Supplier**	**Cat no.**	**Titration**
BUV395	CD8	RPA-T8	BD Biosciences	563795	1 in 250
BUV661	CD4	SK3	BD Biosciences	612962	1 in 50
BUV737	PD-1	EH12.1	BD Biosciences	612791	1 in 50
Aqua (BV510)	Live/Dead		Invitrogen	L34957	1 in 1000
BV570	CD45RA	HI100	Biolegend	304132	1 in 200
BV605	HLA-DR	G46-6	BD Biosciences	562845	1 in 250
BV650	CD69	FN50	Biolegend	310934	1 in 50
BV711	CD25	2A3	BD Biosciences	563159	1 in 50
BV785	CD3	OKT3	Biolegend	317330	1 in 300
Pacific Blue	CD57	HCD57	Biolegend	322316	1 in 600
PE	FoxP3	236A/E7	BD Biosciences	560852	1 in 50
PE-CF594	Granzyme B	GB11	BD Biosciences	562462	1 in 500
PE-Cy5	CD127	A019D5	Biolegend	351324	1 in 100
PE-Cy7	CD56	NCAM16.2	BD Biosciences	335826	1 in 300
AlexaFluor647	Perforin	dG9	Biolegend	308110	1 in 180
AlexaFluor 700	CD16	3G8	BD Biolegend	557920	1 in 300
APC-Cy7	CCR7 (CD197)	G043H7	Biolegend	353212	0.5μl directly to cell pellet

Lymphoid panel

**Fluorochrome**	**Marker**	**Clone**	**Supplier**	**Cat no.**	**Titration**
BUV395	CD14	MφP9	BD	563561	1 in 100
BUV615	CCR2	LS132.1D9	BD	751045	1 in 100
BUV661	HLA-DR	G46-6	BD	612981	1 in 50
BUV737	CD16	3G8	BD	612786	1 in 50
BV421	CD163	GHI/61	Biolegend	333612	1 in 50
BV510	CD3	OKT3	Biolegend	317332	1 in 50
BV510	CD19	HIB19	Biolegend	302242	1 in 50
BV510	CD56	5.1H11	Biolegend	362534	1 in 50
BV510	CD304				1 in 50
BV570	live/dead yellow		Invitrogen	L34968	1 in 1000
BV650	CD31	WM59	BD	740571	1 in 100
BV711	CD141	1A4	BD	563155	1 in 25

BV785	CD55	IA10	BD	742681	1 in 40
BB515	CD206	19.2	BD	564668	1 in 25
BB700	CD1c	F10/21A3	BD	746095	1 in 50
PE-CF594	CD34	563	BD	562449	1 in 200
PECy5	CD5	-	BD	555354	1 in 100
PE-Cy7	CD11c	3.9	Biolegend	301608	1 in 25
AlexaFluor647	CD9	HI9a	Biolegend	312107	1 in 50
AlexaFluor 700	CD45	HI30	BD	560566	1 in 50
APC-Cy7	CD36	GPIIIb	Biolegend	312108	1 in 50
**FAP panel**					
**Fluorochrome**	**Marker**	**Clone**	**Supplier**	**Cat no.**	**Titration**
BV650	CD31	WM59	BD	740571	1 in 100
BV786	CD55	IA10	BD	742681	1 in 40
7-AAD	live/dead		BD	559925	5 µl per sample
PE	CD74	LN2	Biolegend	326807	1 in 20
PE-CF594	CD34	563	BD	562449	1 in 100
AlexaFluor647	EZR		NovusBiologicals	NBP2-52977AF647	1 in 20
AlexaFluor700	CD45	HI30	BD	560566	1 in 100
