# Supplementary material for: An integrated single cell and spatial transcriptomic map of human white adipose tissue
Source: Nat Commun. 2023 Mar 15;14:1438. doi: 10.1038/s41467-023-36983-2 (PMC10017705; doi:10.1038/s41467-023-36983-2)
Supplement: Supplementary file 3 — Description of Additional Supplementary Files [file 41467_2023_36983_MOESM3_ESM.pdf]

Description of Additional Supplementary Files

File Name: Supplementary Data 1
Description: Marker gene lists for each of the 17 data sets used in the present study.

File Name: Supplementary Data 2
Description: Marker gene lists for each class based on the integrated data presented in this study.
