# Supplementary material for: An integrated single cell and spatial transcriptomic map of human white adipose tissue
Source: Nat Commun. 2023 Mar 15;14:1438. doi: 10.1038/s41467-023-36983-2 (PMC10017705; doi:10.1038/s41467-023-36983-2)
Supplement: Supplementary file 6 — Reporting Summary [file 41467_2023_36983_MOESM6_ESM.pdf]

nature portfolio

Corresponding author(s): Niklas Mejhert and Mikael Rydén

Last updated by author(s): Feb 23, 2023

# Reporting Summary

Nature Portfolio wishes to improve the reproducibility of the work that we publish. This form provides structure for consistency and transparency in reporting. For further information on Nature Portfolio policies, see our Editorial Policies and the Editorial Policy Checklist.

## Statistics

For all statistical analyses, confirm that the following items are present in the figure legend, table legend, main text, or Methods section.

| n/a | Confirmed | |
|---|---|---|
| ☐ | ☒ | The exact sample size (*n*) for each experimental group/condition, given as a discrete number and unit of measurement |
| ☐ | ☒ | A statement on whether measurements were taken from distinct samples or whether the same sample was measured repeatedly |
| ☐ | ☒ | The statistical test(s) used AND whether they are one- or two-sided<br>*Only common tests should be described solely by name; describe more complex techniques in the Methods section.* |
| ☒ | ☐ | A description of all covariates tested |
| ☐ | ☒ | A description of any assumptions or corrections, such as tests of normality and adjustment for multiple comparisons |
| ☐ | ☒ | A full description of the statistical parameters including central tendency (e.g. means) or other basic estimates (e.g. regression coefficient) AND variation (e.g. standard deviation) or associated estimates of uncertainty (e.g. confidence intervals) |
| ☐ | ☒ | For null hypothesis testing, the test statistic (e.g. *F*, *t*, *r*) with confidence intervals, effect sizes, degrees of freedom and *P* value noted<br>*Give P values as exact values whenever suitable.* |
| ☒ | ☐ | For Bayesian analysis, information on the choice of priors and Markov chain Monte Carlo settings |
| ☒ | ☐ | For hierarchical and complex designs, identification of the appropriate level for tests and full reporting of outcomes |
| ☒ | ☐ | Estimates of effect sizes (e.g. Cohen's *d*, Pearson's *r*), indicating how they were calculated |

*Our web collection on statistics for biologists contains articles on many of the points above.*

## Software and code

Policy information about availability of computer code

| | |
|---|---|
| Data collection | No software/ code was used to acquire data. |
| Data analysis | All codes for this work is publicly available at https://github.com/lmassier/hWAT_singlecell.<br>Softwares used include:<br>10x Genomics Cell Ranger (v6.0.2)<br> and 10x Genomics Cell Ranger (v<br>.6.0.1)<br>CellBender (v0.2.0)<br>scDblFinder (v1.5.11)<br>R v4.1.2<br>Seurat v4.1.0<br>sctransform (within Seurat)<br>Harmony v0.1.0<br>igraph v1.2.11<br>Cytoscape 3.7.1<br>Rcy3 v2.14.1<br>pdfCluster v1.0-3<br>lisi v1.0<br>kBET v0.99.6<br>sceasy v0.0.6<br>reticulate v1.24 |

RCTD v2.0.0
BisqueRNA v1.0.5
scVI v.0.15.1
meta v5.2-0
FlowJo Software v10.7.1 and v10.8.0
UMAP FlowJo plugin v3.1
FlowJo Phenograph plugin v3
cell2location v0.1
stereoscope v0.2.0
SPOTlight v0.1.0
Tangram v1.0.2
DestVI v0.16.2
CellChat v1.4.0
GraphPad Prism v9

For manuscripts utilizing custom algorithms or software that are central to the research but not yet described in published literature, software must be made available to editors and reviewers. We strongly encourage code deposition in a community repository (e.g. GitHub). See the Nature Portfolio guidelines for submitting code & software for further information.

# Data

Policy information about availability of data

All manuscripts must include a data availability statement. This statement should provide the following information, where applicable:

- Accession codes, unique identifiers, or web links for publicly available datasets
- A description of any restrictions on data availability
- For clinical datasets or third party data, please ensure that the statement adheres to our policy

All data retrospectively analyzed or generated are available here: https://doi.org/10.17632/y3pxvr4xbf.1 (snSeq data), https://github.com/lmassier/hWAT_singlecell (code, source data, FACS raw data, Cell Typist models) and a source data file was generated.
Parts of the data analyzed in this work were download from the indicated studies, references and accsession numbers are detailed in Extended Data Tables 1 and 3.
For single nuclei (snSeq) and single cell sequencing (scSeq), data was downloaded from the following publications/ repositories:
Sun et al. (snSeq), E-MTAB-9199, Nature 587, 98-102 (2020)
Hildreth et al. (scSeq) , GSE155960, GSE156110, Nat Immunol 22, 639-653 (2021)
Vijay et al. (scSeq), Nat Metab 2, 97-109 (2020)
Acosta et al. (scSeq), Communication with author, Stem Cell Res Ther 8, 250 (2017)
Merrick et al. (scSeq),GSE128889, Science 364(2019)
Karunakaran et al. (scSeq), GSE151889, Nat Metab 2, 1113-1125 (2020)
Emont et al. (snSeq, scSeq), E-MTAB-6677, GSE135134, GSE128889, Nature 603, 926-933 (2022)
Jaitin et al. (scSeq), GSE128518, Cell 178, 686-698 e614 (2019)
Angueira et al. (snSeq), GSE164528 Nat Metab 3, 469-484 (2021)
Spatial data was produced by our lab and recently published: Bäckdahl & Franzén et al. (Cell Metab. 2021 Nov 2;33(11):2301).

Included clinical cohorts with available bulk RNA sequencing or array data of white adipose tissue are:
Arner, E. et al. Microarray GeneChip Human Gene 1.0 ST Array, GSE25402 Diabetes 61, 1986-1993 (2012)
Arner, P. et al. Microarray Clariom D array, GSE113080 Cell Metabolism 28, 45-54 (2018)
Kerr, A. et al. Microarray Clariom D array, GSE199063 J Intern Med 288, 219-233 (2020)
Arner, P. et al. Microarray GeneChip Human Transcriptome Array 2.0, GSE76399 Diabetologia 59, 2393-2405 (2016)
Petrus, P. et al. Microarray Affymetrix Human Gene 1.1 ST array, GSE59034 Cell Rep 25, 551-560 (2018).
Krieg, L et al. Microarray HumanHT - 12 v4, Suppl. Table 7 Gut 71, 2179-2193 (2022).
Imbert, A. et al. RNASeq HiSeq 2500, GSE141221 J Clin Endocrinol Metab 107, e130-e142 (2022).
Armenise, C. et al. RNASeq HiSeq 2000, GSE95640 Am J Clin Nutr 106, 736-746 (2017).

Adipocyte specific genes were identified using the FANTOM5 data base (https://fantom.gsc.riken.jp/5/)

Additional data that support the findings of this study are available from the corresponding authors upon reasonable request.

# Human research participants

Policy information about studies involving human research participants and Sex and Gender in Research.

| | |
|---|---|
| Reporting on sex and gender | The number of males and females are indicated where information was available. |
| Population characteristics | Information on the clinical cohorts are given in Figures 1 and 8. |
| Recruitment | Generation and analysis of new data was done retrospectively and no new subjects were recruited for this publication. |
| Ethics oversight | The study was performed in agreement with the Declaration of Helsinki and approved by the Ethics Committee of the University of Leipzig (approval numbers: 159-12-21052012 and 004/21-ek) and the Stockholm regional board of ethics (Clinical trials identifiers; NCT01785134, NCT01727245). All patients provided written informed consent before participation. |

Note that full information on the approval of the study protocol must also be provided in the manuscript.

# Field-specific reporting

Please select the one below that is the best fit for your research. If you are not sure, read the appropriate sections before making your selection.

☒ Life sciences ☐ Behavioural & social sciences ☐ Ecological, evolutionary & environmental sciences

For a reference copy of the document with all sections, see nature.com/documents/nr-reporting-summary-flat.pdf

# Life sciences study design

All studies must disclose on these points even when the disclosure is negative.

| | |
|---|---|
| Sample size | This work reports a meta-analysis and all details on the studies are given in the present work as well as in the original references. No sample size calculations were performed. All identified cohorts (with the exception of Hildreth et al.) were included alongside 4 newly generated snSeq cohorts.<br>Imaging experiments were repeated multiple time (n>5) on independent samples with identical results. Flow cytometry validation was performed in samples of 10 subjects across a wide range of BMI to validate findings identified in the meta-analysis. |
| Data exclusions | Statistical analyses are described in detail in the Methods section. One study (Hildreth et al) was excluded as described in the manuscript. |
| Replication | The manuscript represents a meta-analysis, combining results from 14 different cohorts. The Result/Discussion sections highlight findings that were identified in all cohorts as well as findings that could not be identified/ replicated in multiple cohorts. |
| Randomization | All newly analyzed data was generated retrospectively and there are no treatment/ control groups, therefore randomization could not be performed |
| Blinding | All newly analyzed data was generated retrospectively and there are no treatment/ control groups, therefore blinding could not be performed |

# Reporting for specific materials, systems and methods

We require information from authors about some types of materials, experimental systems and methods used in many studies. Here, indicate whether each material, system or method listed is relevant to your study. If you are not sure if a list item applies to your research, read the appropriate section before selecting a response.

## Materials & experimental systems

| n/a | Involved in the study | |
|---|---|---|
| ☐ | ☒ | Antibodies |
| ☒ | ☐ | Eukaryotic cell lines |
| ☒ | ☐ | Palaeontology and archaeology |
| ☒ | ☐ | Animals and other organisms |
| ☐ | ☒ | Clinical data |
| ☒ | ☐ | Dual use research of concern |

## Methods

| n/a | Involved in the study | |
|---|---|---|
| ☒ | ☐ | ChIP-seq |
| ☐ | ☒ | Flow cytometry |
| ☒ | ☐ | MRI-based neuroimaging |

## Antibodies

| | |
|---|---|
| Antibodies used | Primary antibodies used for protein detection by immunofluorescence were anti-TREM2 (Proteintech Cat#13483-1-AP, 1:100), anti-CD9 (Proteintech Cat#60232-1-Ig, 1:500), anti-SLIT2 (Proteintech Cat#20217-1-AP, 1:200), and anti-CD31 (Agilent Technologies Cat#M082329-2, 1:100). Secondary antibodies were donkey anti-rabbit conjugated with Alexa Fluor 594 (Thermofisher Cat#A-21207, 1:200) and donkey anti-mouse conjugated with Alexa Flour 488 (Thermofisher Cat#A-21202, 1:200).<br>Antibodies used for flow cytometry were as follows: anti-CD8-BUV395 (BD Cat#563795, 1:250), anti-CD4-BUV661 (BD Cat#612962, 1:50), anti-PD1-BUV737 (BD Cat#612791, 1:50), anti-CD45RA-BV570 (Biolegend Cat#304132, 1:200), anti-HLA-DR-BV605 (BD Cat#562845, 1:250), anti-CD69-BV650 (Biolegend Cat#304132, 1:50), anti-CD25-BV711 (BD Cat#563159, 1:50), anti-CD3-BV785 (Biolegend Cat#317330, 1:300), anti-CD57-PB (Biolegend Cat#322316, 1:600), anti-FoxP3-PE (BD Cat#560852, 1:50), anti-GranzymeB-PE-CF594 (BD Cat#335826, 1:500), anti-CD127-PE-Cy5 (Biolegend Cat#351324, 1:100), antiCD56-PE-Cy7 (BD Cat#335826, 1:300), anti-Perforin-AF647 (Biolegend Cat#308110, 1:180), anti-CCR7-APC-Cy7 (Biolegend Cat#353212, 1:20), anti-CD14-BUV395 (BD Cat#563561, 1:100), anti-CCR2-BUV615 (BD Cat#751045, 1:100), anti-HLA-DR-BUV661 (BD Cat#612981, 1:50), anti-CD16-BUV737 (BD Cat# 612786, 1:50), anti-CD163-BV421 (Biolegend Cat#333612, 1:50), anti-CD3-BV510 Biolegend Cat#317332, 1:50), anti-CD19-BV510 (Biolegend Cat#302242, 1:50), anti-CD56-BV510 (Biolegend Cat#362534, 1:50), anti-CD304-BV510 (Biolegend Cat#743129, 1:50), anti-CD31-BV650 (BD Cat#740571, 1:100), anti-CD141-BV711 (BD Cat#563155, 1:25), anti-CD55-BV785 (BD Cat#742681, 1:40), anti-CD206-BB515 (BD Cat#564668, 1:25), anti-CD1c-BB700 (BD Cat#746095, 1:50), anti-CD34-PE-CF594 (BD Cat#562449, 1:200), anti-CD5-Pe-Cy5 (BD Cat#555354, 1:100), anti-CD11c-PE-Cy7 (Biolegend Cat#301608, 1:25), anti-CD9-AF647 (Biolegend Cat#312107, 1:50), anti-CD45-AF700 (BD Cat#560566, 1:50), anti-CD36-APC-Cy7 (Biolegend Cat#312108, 1:50), anti-CD74-PE (Biolegend Cat#326807, 1:20), and anti-EZR-AF647 (Novus Biologicals Cat#NBP2-52977AF647, 1:20). |

Validation

All antibodies used in the study are commercially available and validated by the suppliers and/or widely used in other publications.
The TREM2 antiobody has been KD/KO validated. Positive IHC detected in mouse brain tissue. Manufacturer listed citations (12) can be accessed at https://www.ptglab.com/products/TREM2-Antibody-13483-1-AP.htm#tested-applications.
The CD9 antibody has been KD/KO validated. Positive IHC detected in human ovary tumor tissue, human colon cancer tissue, human tonsillitis tissue, human breast cancer tissue. Manufacturer listed citations (46) can be accessed at https://www.ptglab.com/products/CD9-Antibody-60232-1-Ig.htm.
The SLIT2 antibody has been KD/KO validated. Positive IHC detected in human kidney tissue, human breast cancer tissue. Citations (14) can be accessed at https://www.ptglab.com/products/SLIT2-Specific-Antibody-20217-1-AP.htm.
The CD31 (JC70A) antibody was clustered as anti-CD31 at the Fifth International Workshop and Conference on Human Leucocyte Differentiation Antigens, and the epitope recognized was found to be within the extracellular domain 1 according to the manufacturer. CItations (3) can be accessed at https://www.agilent.com/en/product/immunohistochemistry/antibodies-controls/primary-antibodies/cd31-endothelial-cell-%28concentrate%29-76539#productdetails.
All flow cytometry antibodies were widely established clones used at a manufacturer-recommended concentration or titrated in-house.
The CD8 (RPA-T8) antibody specifically binds to CD8 alpha (CD8α) and is recommended for routine use in flow cytometry by the manufacturer. Development references (3) can be accessed at https://www.bdbiosciences.com/en-nz/products/reagents/flow-cytometry-reagents/research-reagents/single-color-antibodies-ruo/buv395-mouse-anti-human-cd8.563795.
The CD4 (SK3) antibody specifically binds to CD4 and is routinely tested for flow cytometry. Development references (6) can be accessed at https://www.bdbiosciences.com/en-nz/products/reagents/flow-cytometry-reagents/research-reagents/single-color-antibodies-ruo/buv661-mouse-anti-human-cd4.612962.
The PD-1 (EH12.1) antibody specifically binds to CD279 and is routinely tested for flow cytometry. Development references (8) can be accessed at https://www.bdbiosciences.com/en-nz/products/reagents/flow-cytometry-reagents/research-reagents/single-color-antibodies-ruo/buv737-mouse-anti-human-cd279-pd-1.612791.
The CD45RA (HI100) antibody is quality control tested by immunofluorescent staining with flow cytometric analysis. Product citations (8) can be accessed at https://www.biolegend.com/en-gb/cellular-dyes-and-ancillary-products/brilliant-violet-570-anti-human-cd45ra-antibody-7365.
The HLA-DR (G46-6) antibody specifically binds to HLA-DR and is routinely tested for flow cytometry. Development references can be accessed at https://www.bdbiosciences.com/en-au/products/reagents/flow-cytometry-reagents/research-reagents/single-color-antibodies-ruo/bv605-mouse-anti-human-hla-dr.562845.
The CD69 (FN50) antibody is quality control tested by immunofluorescent staining with flow cytometric analysis. Product citations (7) can be accessed at https://www.biolegend.com/de-at/products/brilliant-violet-650-anti-human-cd69-antibody-8345.
The CD25 (2A3) monoclonal antibody specifically binds to human CD25, the low-affinity alpha subunit of the Interleukin-2 Receptor (IL-2R α ) and is routinely used in flow cytometry. Development references (13) can be accessed at https://www.bdbiosciences.com/en-us/products/reagents/flow-cytometry-reagents/research-reagents/single-color-antibodies-ruo/bv711-mouse-anti-human-cd25.563159.
The CD3 (OKT3) antibody is quality control tested by immunofluorescent staining with flow cytometric analysis. Product citations (7) can be accessed at https://www.biolegend.com/en-gb/neuroscience-1/brilliant-violet-785-anti-human-cd3-antibody-7977.
The CD57 (HCD57) antibody is quality control tested for flow cytometry. It is referenced 25 times in citeab (https://www.citeab.com/antibodies/522739-322316-pacific-blue-anti-human-cd57-antibody).
The FoxP3 (The 236A/E7) antibody reacts with the human FoxP3 transcription factor and is recommended for routine use by flow cytometry. Development references (8) can be accessed at https://www.bdbiosciences.com/en-au/products/reagents/flow-cytometry-reagents/research-reagents/single-color-antibodies-ruo/pe-mouse-anti-human-foxp3.560852.
The granzyme B (GB11) antibody specifically reacts with human granzyme B and is recommended for routine use by flow cytometry. Development references (6) can be accessed at https://www.bdbiosciences.com/en-au/products/reagents/flow-cytometry-reagents/research-reagents/single-color-antibodies-ruo/pe-cf594-mouse-anti-human-granzyme-b.562462.
The CD127 (A019D5) antibody is quality control tested by immunofluorescent staining with flow cytometric analysis. Product citations (1) can be accessed at https://www.biolegend.com/fr-ch/clone-search/pe-cyanine5-anti-human-cd127-il-7ralpha-antibody-7504.
The CD56 (NCAM16.2) antibody is extensively validated and tested for reproducibility and repeatability using flow cytometry and cited 15 times according to the manufacturer. The citations can be accessed at https://www.bdbiosciences.com/en-fi/products/reagents/flow-cytometry-reagents/clinical-diagnostics/single-color-antibodies-asr-ivd-ce-ivd/cd56-pe-cy-7.335826.
The perforin (dG9) antibody primarily recognizes perforin associated with cytotoxic granules and is recommended for flow cytometry according to the manufacturer. Product citations (16) can be accessed at https://www.biolegend.com/de-de/products/alexa-fluor-647-anti-human-perforin-antibody-3156.
The CD16 (3G8) antibody specifically recognizes CD16a and CD16b, low-affinity receptors for the Fc region of IgG. Development references (5) can be accessed at https://www.bdbiosciences.com/en-us/products/reagents/flow-cytometry-reagents/research-reagents/single-color-antibodies-ruo/alexa-fluor-700-mouse-anti-human-cd16.557920.
The CCR7 (G043H7) antibody is quality control tested by immunofluorescent staining with flow cytometric analysis. Product citations (31) can be accessed at https://www.biolegend.com/nl-be/explore-new-products/apc-cyanine7-anti-human-cd197-ccr7-antibody-7524.
The CD14 (MΦP9) antibody specifically binds to CD14 and is used routinely in flow cytometry. Development references (6) can be accessed at https://www.bdbiosciences.com/en-fi/products/reagents/flow-cytometry-reagents/research-reagents/single-color-antibodies-ruo/buv395-mouse-anti-human-cd14.563561.
The CCR2 (LS132.1D9) antibody specifically recognizes C-C chemokine receptor type 2. Development references (3) can be accessed at https://www.bdbiosciences.com/en-fi/products/reagents/flow-cytometry-reagents/research-reagents/single-color-antibodies-ruo/buv615-mouse-anti-human-ccr2-cd192.751045.
The HLA-DR (G46-6) antibody specifically binds to HLA-DR and is used routinely in flow cytometry. Development references (5) can be accessed at https://www.bdbiosciences.com/en-fi/products/reagents/flow-cytometry-reagents/research-reagents/single-color-antibodies-ruo/buv661-mouse-anti-human-hla-dr.612981.
The CD16 (3G8) monoclonal antibody specifically recognizes CD16a and CD16b, low-affinity receptors for the Fc region of IgG. It is used routinely in flow cytometry. Development references (10) can be accessed at https://www.bdbiosciences.com/en-fi/products/reagents/flow-cytometry-reagents/research-reagents/single-color-antibodies-ruo/buv737-mouse-anti-human-cd16.612786.
The CD163 (GHI/61) antibody binds to domain 7 of CD163 and is recommended for flow cytometry according to the manufacturer. Product citations (6) can be accessed at https://www.biolegend.com/fr-fr/products/brilliant-violet-421-anti-human-cd163-antibody-8810.

The CD19 (HIB19) antibody is recommended for flow cytometry and partially blocks anti-human CD19 clones 4G7 and SJ25C1 staining based on manufacturer's in-house testing. Product citations (29) can be accessed at https://www.biolegend.com/en-gb/products/brilliant-violet-510-anti-human-cd19-antibody-8004.
The CD56 (5.1H11) antibody is quality control tested by immunofluorescent staining with flow cytometric analysis. Product citations (2) can be accessed at https://www.biolegend.com/en-gb/products/brilliant-violet-510-anti-human-cd56-ncam-antibody-11834.
The CD304 (U21-1283) monoclonal antibody specifically recognizes Neuropilin-1 (NRP1) and is recommended for flow cytometry. Development references (6) can be accessed at https://www.bdbiosciences.com/en-se/products/reagents/flow-cytometry-reagents/research-reagents/single-color-antibodies-ruo/bv510-mouse-anti-human-neuropilin-1-cd304.743129.
The FACS-CD31 (WM59) antibody specifically binds to platelet endothelial cell adhesion molecule-1 and is recommended for flow cytometry. Development references (6) can be accessed at https://www.bdbiosciences.com/en-se/products/reagents/flow-cytometry-reagents/research-reagents/single-color-antibodies-ruo/bv650-mouse-anti-human-cd31.740571.
The CD141 (1A4) monoclonal antibody specifically binds to CD141 and is recommended for flow cytometry. Development references (3) can be accessed at https://www.bdbiosciences.com/en-se/products/reagents/flow-cytometry-reagents/research-reagents/single-color-antibodies-ruo/bv711-mouse-anti-human-cd141.563155.
The CD55 (IA10) antibody specifically binds to CD55 and is recommended for flow cytometry. Development references (5) can be accessed at https://www.bdbiosciences.com/en-se/products/reagents/flow-cytometry-reagents/research-reagents/single-color-antibodies-ruo/bv786-mouse-anti-human-cd55.742681.
The CD206 (19.2) antibody specifically binds to CD206 and is recommended for flow cytometry. Development references (5) can be accessed at https://www.bdbiosciences.com/en-se/products/reagents/flow-cytometry-reagents/research-reagents/single-color-antibodies-ruo/bb515-mouse-anti-human-cd206.564668.
The CD1c (F10/21A3) antibody specifically binds to CD1c and is recommended for flow cytometry. Development references (5) can be accessed at https://www.bdbiosciences.com/en-se/products/reagents/flow-cytometry-reagents/research-reagents/single-color-antibodies-ruo/bb700-mouse-anti-human-cd1c.746095.
The CD34 (563) antibody specifically binds to CD34 and is recommended for flow cytometry. Development references (6) can be accessed at https://www.bdbiosciences.com/en-se/products/reagents/flow-cytometry-reagents/research-reagents/single-color-antibodies-ruo/pe-cf594-mouse-anti-human-cd34.562449.
The CD5 (UCHT2) antibody specifically binds to CD5 and is recommended for flow cytometry. Development references (6) can be accessed at https://www.bdbiosciences.com/en-se/products/reagents/flow-cytometry-reagents/research-reagents/single-color-antibodies-ruo/pe-cy-5-mouse-anti-human-cd5.555354.
The CD11c (3.9) antibody preferentially binds the activated form of CD11c, is specific for the I domain of CD11c, and is able to partially block the binding of CD11c and ICAM-4. 3.9 binding is divalent cation dependent according to the manufacturer. Product citations (12) can be accessed at https://www.biolegend.com/nl-be/clone-search/pe-cyanine7-anti-human-cd11c-antibody-2803?GroupID=BLG7890.
The FACS-CD9 (HI9a) antibody is quality control tested by immunofluorescent staining with flow cytometric analysis and cited once according to the manufacturer (https://www.biolegend.com/nl-be/products/apc-anti-human-cd9-antibody-15072).
The CD45 (HI30) antibody specifically binds to the 180, 190, 205, 220 kDa protein isoforms of CD45 and is recommended for flow cytometry. Development references (13) can be accessed at https://www.bdbiosciences.com/en-au/products/reagents/flow-cytometry-reagents/research-reagents/single-color-antibodies-ruo/alexa-fluor-700-mouse-anti-human-cd45.560566.
The CD36 (GPIIIb) antibody is quality control tested by immunofluorescent staining with flow cytometric analysis and cited once according to the manufacturer (https://www.biolegend.com/nl-be/products/apc-anti-human-cd9-antibody-15072).
The CD74 (LN2) antibody is reactive with an epitope residing within 60 amino acids of the extracytoplasmic, COOH terminus of the protein, and is recommended for flow cytometry. Product citations (8) can be accessed at https://www.biolegend.com/nl-be/products/pe-anti-human-cd74-antibody-4093.
Validated applications for the ezrin antibody include immunocytochemistry/immunofluorescence, immunohistochemistry, immunohistochemistry-paraffin and western blot. The conjugated antibody was titrated for flow cytometry in-house and represented expected differences between omental and subcutaneous adipose tissue samples.
The TREM2 antibody is KD/KO validated. Positive IHC detected in mouse brain tissue. 12 citations. It can be accessed at https://www.ptglab.com/products/TREM2-Antibody-13483-1-AP.htm#tested-applications
The IF CD9 antibody is KD/KO validated. Positive IHC detected in human ovary tumor tissue, human colon cancer tissue, human tonsillitis tissue, human breast cancer tissue. 46 citations. It can be accessed at: https://www.ptglab.com/products/CD9-Antibody-60232-1-Ig.htm
The SLIT2 antibody os KD/KO validated. Positive IHC detected in human kidney tissue, human breast cancer tissue. 14 citations. It can be accessed at: https://www.ptglab.com/products/SLIT2-Specific-Antibody-20217-1-AP.htm
The IF CD31 antibody is a monoclonal antibody with clone number : JC70A and code number : M0823; The antibody was clustered as anti-CD31 at the Fifth International Workshop and Conference on Human Leucocyte Differentiation Antigens. The epitope recognized was found to be within the extracellular domain 1.
In Western blotting of membrane preparations from a spleen rich in the antigen or from normal platelets, the antibody labels bands of respectively 100 kDa and 130 kDa, the latter corresponding to classic CD31. The smaller band of 100 kDa observed with the splenic preparation may be due to proteolytic breakdown or to variations in glycosylation
3 references. It can be accessed at https://www.agilent.com/en/product/immunohistochemistry/antibodies-controls/primary-antibodies/cd31-endothelial-cell-%28concentrate%29-76539#productdetails

## Clinical data

Policy information about clinical studies
All manuscripts should comply with the ICMJE guidelines for publication of clinical research and a completed CONSORT checklist must be included with all submissions.

Clinical trial registration: Primary outcomes for both clinical trials retrospectively analyzed have been described at clinicaltrials.gov (NCT01785134 (DEOSH) and NCT01727245 (NEFA)) and the studies have been completed.

Study protocol: Study protocols can be obtained from clinicaltrials.gov under the following links: https://clinicaltrials.gov/ct2/show/NCT01785134 and https://clinicaltrials.gov/ct2/show/NCT01727245

Data collection: Details of data collections can be found under the following links: https://clinicaltrials.gov/ct2/show/results/NCT01785134 and

Data collection

https://clinicaltrials.gov/ct2/show/NCT01727245. Briefly, for NCT01785134 81 participants were recruited, of which 41 in the control group and 40 in the Omentectomy group and data was collected at baseline and 2 years after surgery in the years 2013 to 2015. For NCT01727245, 500 participants were recruited starting in 2011 and the study was finished in 2019.

Outcomes

For NCT01785134 the primary outcome was insulin sensitivity two years after surgery as measured by hyperinsulinemic euglycemic clamp and secondary outcomes were body composition, blood pressure, body mass index and blood lipids at the same time point. All outcomes were previously published:

van der Kolk BW, Muniandy M, Kaminska D, Alvarez M, Ko A, Miao Z, Valsesia A, Langin D, Vaittinen M, Paakkonen M, Jokinen R, Kaye S, Heinonen S, Virtanen KA, Andersson DP, Mannisto V, Saris WH, Astrup A, Ryden M, Blaak EE, Pajukanta P, Pihlajamaki J, Pietilainen KH. Differential Mitochondrial Gene Expression in Adipose Tissue Following Weight Loss Induced by Diet or Bariatric Surgery. J Clin Endocrinol Metab. 2021 Apr 23;106(5):1312-1324. doi: 10.1210/clinem/dgab072.
Andersson DP, Thorell A, Lofgren P, Wiren M, Toft E, Qvisth V, Riserus U, Berglund L, Naslund E, Bringman S, Thorne A, Arner P, Hoffstedt J. Omentectomy in addition to gastric bypass surgery and influence on insulin sensitivity: a randomized double blind controlled trial. Clin Nutr. 2014 Dec;33(6):991-6. doi: 10.1016/j.clnu.2014.01.004. Epub 2014 Jan 12.

For NCT01727245 the primary outcome was insulin sensitivity, adipose tissue function and gene function two years after bariatric surgery. Secondary outcomes included body composition and vascular function. All outcomes were previously published:

Mileti E, Kwok KHM, Andersson DP, Mathelier A, Raman A, Backdahl J, Jalkanen J, Massier L, Thorell A, Gao H, Arner P, Mejhert N, Daub CO, Ryden M. Human White Adipose Tissue Displays Selective Insulin Resistance in the Obese State. Diabetes. 2021 Jul;70(7):1486-1497. doi: 10.2337/db21-0001. Epub 2021 Apr 16.
Backdahl J, Andersson DP, Eriksson-Hogling D, Caidahl K, Thorell A, Mileti E, Daub CO, Arner P, Ryden M. Long-Term Improvement in Aortic Pulse Wave Velocity After Weight Loss Can Be Predicted by White Adipose Tissue Factors. Am J Hypertens. 2018 Mar 10;31(4):450-457. doi: 10.1093/ajh/hpx201

# Flow Cytometry

## Plots

Confirm that:

☒ The axis labels state the marker and fluorochrome used (e.g. CD4-FITC).

☒ The axis scales are clearly visible. Include numbers along axes only for bottom left plot of group (a 'group' is an analysis of identical markers).

☒ All plots are contour plots with outliers or pseudocolor plots.

☒ A numerical value for number of cells or percentage (with statistics) is provided.

## Methodology

| | |
|---|---|
| Sample preparation | Described in detail in the original reference (#59) |
| Instrument | BD LSRFortessa analyzer equipped with 355, 405, 488, 561 and 640 nm lasers and DIVA software (BD Biosciences) |
| Software | FlowJo Software v10.8.0 (BD Biosciences). |
| Cell population abundance | Samples were not sorted in this study. |
| Gating strategy | Forward and side scatter-area parameters were used to gate the target cell population. Subsequently, SSC-A/SSC-H, FSC-W/FSC-H, or FSC-A/FSC-H gates were applied to select only single cells. Dead cells were removed via gating on live/dead dye-negative population. Subsequent gates were set based on fluorescence minus one (FMO) controls or an unstained control as displayed in figures or figure legends. |

☒ Tick this box to confirm that a figure exemplifying the gating strategy is provided in the Supplementary Information.
